# The Norwegian Stroke in the Young Study (NOR-SYS): Rationale and design

**DOI:** 10.1186/1471-2377-13-89

**Published:** 2013-07-17

**Authors:** Annette Fromm, Lars Thomassen, Halvor Naess, Rudy Meijer, Geir Egil Eide, Jostein Kråkenes, Christian A Vedeler, Eva Gerdts, Terje H Larsen, Karel K-J Kuiper, Elin Laxdal, David Russell, Turgut Tatlisumak, Ulrike Waje-Andreassen

**Affiliations:** 1 Department of Neurology, Haukeland University Hospital, Bergen, Norway; 2 Department of Clinical Medicine, University of Bergen, Bergen, Norway; 3 Julius Center of Health Sciences and Primary Care, University Medical Center Utrecht, Utrecht, The Netherlands; 4 Centre for Clinical Research, Haukeland University Hospital, Bergen, Norway; 5 Department of Public Health and Primary Health Care, Lifestyle Epidemiology Research Group, University of Bergen, Bergen, Norway; 6 Department of Radiology, Haukeland University Hospital, Bergen, Norway; 7 Department of Heart Disease, Haukeland University Hospital, Bergen, Norway; 8 Department of Clinical Science, University of Bergen, Bergen, Norway; 9 Department of Biomedicine, University of Bergen, Bergen, Norway; 10 Department of Vascular Surgery, Haukeland University Hospital, Bergen, Norway; 11 Department of Neurology, Cerebrovascular Centre, Oslo University Hospital, Rikshospitalet, Oslo, Norway; 12 Institute of Clinical Medicine, University of Oslo, Oslo, Norway; 13 Department of Neurology, Helsinki University Central Hospital, Helsinki, Finland

**Keywords:** Ischemic stroke, Stroke in the young, Atherosclerosis, Arterial disease, Ultrasound, Heredity, Vascular risk, Long-term outcome, Mortality

## Abstract

**Background:**

Ischemic stroke in young adults is a major health problem being associated with a higher vascular morbidity and mortality compared to controls, and a stroke recurrence rate of 25% during the first decade. The assumed cause of infarction and the detected risk factors determine the early- and long-term treatment. However, for many patients the cause of stroke remains unknown. Risk factor profile and etiology differ in young and elderly ischemic stroke patients, and atherosclerosis is the determined underlying condition in 10 to 15%. However, subclinical atherosclerosis is probably more prevalent and may go unrecognized.

Ultrasound imaging is a sensitive method for the detection of arterial disease and for measurement of adipose tissue. The relationship between intima-media thickness (IMT), plaques, cardiovascular risk factors including visceral adipose tissue (VAT) and ischemic events has repeatedly been shown.

We have established The Norwegian Stroke in the Young Study (NOR-SYS) as a three-generation research program with the goal to increase our knowledge on heredity and the development of arterial disease and ischemic stroke. Extended standardized ultrasound examinations are done in order to find subclinical vessel disease for early and better prophylaxis.

**Methods/Design:**

NOR-SYS is a prospective long-term research program. Standardized methods are used for anamnestic, clinical, laboratory, imaging, and ultrasound data collection in ischemic stroke patients aged ≤60 years, their partners and joint adult offspring. The ultrasound protocol includes the assessment of intracranial, carotid and femoral arteries, abdominal aorta, and the estimation of VAT. To date, the study is a single centre study with approximately 400 patients, 250 partners and 350 adult offspring expected to be recruited at our site.

**Discussion:**

NOR-SYS aims to increase our knowledge about heredity and the development of arterial vascular disease in young patients with ischemic stroke and their families. Moreover, optimization of diagnostics, prophylaxis and early intervention are major targets with the intention to reduce stroke recurrence and other clinical arterial events, physical disability, cognitive impairment and death.

NOR-SYS is reviewed and approved by the Regional Committee for Medical and Health Research Ethics, Western-Norway (REK-Vest 2010/74), and registered in ClinicalTrials.gov: NCT01597453.

## Background

Cerebrovascular and coronary artery disease are the main causes of disability and death in the western world [1]. According to observational studies where TOAST criteria have been used, atherosclerosis is the underlying condition in 10 to 15% of patients with ischemic cerebrovascular events of determined etiology [2]. However, in 30-40% of cases the cause of stroke remains unknown [3]. Risk factor profile and etiology differ in young ischemic stroke patients compared to the elderly [3-5]. In addition, young patients have a higher vascular morbidity and mortality compared to healthy controls [6-9], and recurrent ischemic events are common [10,11]. Further, a significant portion of ischemic stroke patients have unrecognized atherosclerosis not only located to cervical arteries, but as well to intracranial arteries [12], to coronary arteries [13], to the aortic arch [14] and to femoral arteries [15]. As therapeutic options are limited, primary and secondary prophylaxis of atherosclerosis and generalized arterial disease should be a major target with the purpose to reduce long-term disability and death among young stroke patients.

Ultrasound imaging is a sensitive, non-invasive, and low-cost method for the detection of arterial vessel disease in major arteries [16,17]. The measurement of carotid intima-media thickness (cIMT) and plaques in B-mode ultrasound has become a tool for vascular risk prediction, as the relationship between IMT, plaques, cardiovascular risk factors and future ischemic events has consequently been shown in several longitudinal studies, predominantly in older individuals [17-27]. However, the value of IMT measurements in all carotid artery segments compared with measurements in the distal CCA alone is disputable [28], and a recent meta-analysis concluded that cIMT measurements in the CCA alone adds little to the improvement of a 10-year risk prediction [29].

NOR-SYS is a concept for the standardized gathering of anamnestic, clinical and biological data in young ischemic stroke patients, their partners, and their family members. The intention is to estimate the presence of arterial vessel disease, to determine the individual’s vascular risk profile, and to offer optimal prevention.

Inclusion of the patients’ partners and joint adult offspring is providing a platform for primary vascular prevention and early intervention. Stroke is a result of multifactorial causes with genetic, environmental and life-style components [30]. The combination of a standardized case-history, standardized ultrasound protocols, and a prospective long-term follow-up schedule is expected to give knowledge regarding heredity and vascular co-morbidity. The optimal goal and the major purpose of the study is to reduce vascular morbidity, disability, cognitive impairment and mortality in young ischemic stroke patients.

## Methods and design

**Figure 1:**
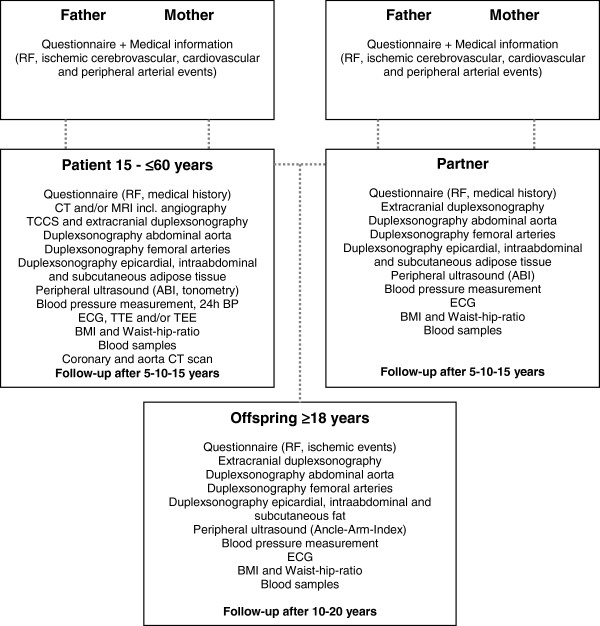
**NOR-SYS Assessment chart.** RF = Risk factors; CT = Computed tomography; MRI = Magnetic resonance imaging; TCCS = Transcranial color-coded sonography; ABI = Ankle-Brachial Index; ECG = Electrocardiogram; TTE = Transthoracic echocardiography, TEE = Transesophageal echocardiography; 24 h BP = 24 hour blood pressure monitoring; BMI = Body Mass Index.

NOR-SYS is intended to be a national multicenter study, performed by co-operating neurological departments in Norway. The study was initiated at Haukeland University Hospital, Bergen, in September 2010. The inclusion period will be 5 years. NOR-SYS is designed as a three-generation study with prospective long-term follow-up design. In addition to a routine cerebro-cardiovascular work-up including clinical examination, neuroimaging, cardiac investigations, and laboratory analyses, all participating patients and relatives are investigated according to the NOR-SYS protocol (Figure 1). This includes questionnaires regarding vascular disease burden in the family, the patient’s medical history and life styles. In addition, all patients are examined by transcranial, extracranial, abdominal and peripheral ultrasound, arterial stiffness measurements, and 24 hour blood pressure monitoring. Participants with undocumented but suspected coronary and/or peripheral arterial disease are referred to the Department of Cardiology and the Department of Vascular Surgery, respectively, for further appropriate investigations, including cardiac computertomography-angiography (CCTA) and CT of the thoracic aorta.

Anthropometric variables, such as height, weight, and waist-hip ratio are measured and EDTA-blood and serum samples are collected to a biobank. The patients’ partners and biological offspring aged ≥18 years are being offered investigations as shown in Figure 1. Data on medical history and life styles are collected from the patients’ biological parents, partners, and offspring by standardized questionnaires. For deceased first-degree relatives, the patient will be asked about their cardio-vascular clinical events and the achieved information will be verified by medical records and data from the Norwegian Cause of Death Registry.

NOR-SYS will be carried out in two phases. In the first, cross-sectional phase, a comprehensive stroke data base on vascular risk factors, arterial ischemic events, and clinical and subclinical atherosclerotic disease burden in the study population is being established.

The second, longitudinal phase will constitute long-term follow-ups, at 5, 10 and 15 years from the time of inclusion for patients and their partners, and at 10 and 20 years from time of inclusion for offspring. The purpose of the follow-up is to observe the biological development of atherosclerosis and vascular disease over time, and to optimize primary and secondary medical prophylaxis. The complete work-up is shown in Figure 1.

### Subject selection

Study participation is offered to all patients with Norwegian residency aged 15 to 60 years, with radiologically documented acute cerebral infarction. All study participation is based on informed written consent. Patients of non-western European ethnicity are investigated in agreement with the NOR-SYS protocol, but are not included in statistical study analyses. Patients with ischemic stroke due to a traumatic cause or subarachnoidal bleeding are excluded from study participation. Spouses and partners of included patients are offered participation as control persons and as reference persons to participating joint offspring. Parents of patients and partners are invited to return standardized questionnaires. All participants are asked for permission to review their relevant medical records from hospitals, specialists or general practitioners for verification.

### Baseline procedures at study inclusion

A. Oral and written study information and signing of informed written consent

B. Anamnestic data collection by standardized questionnaires, including socio-demographic variables, history of previous vascular disease, history of vascular risk factors, data on life styles and nutrition habits, history of other disease, allergies, recent infections, current medication, and circumstances around stroke onset.

C. Neurosonology and Duplex/Doppler ultrasound examinations. Duplex sonography studies are performed using a iU22 Philips Medical Systems, Bothell, WA, USA. Neurosonologists are trained and certified by the most experienced neurosonologists at Haukeland University Hospital (LT and UWA) in collaboration with the University Medical Centre of Utrecht (RM), The Netherlands. Intra- and interobserver variability investigations are performed. Continuous ECG monitoring during the ultrasound examination is done in order to perform standardized carotid and femoral IMT-measurements in the enddiastolic phase of the cardiac cycle. Except for cardiac ultrasound examination, the subject is being placed in supine position.

a) *Transcranial color-coded sonography (TCCS)*. The intracranial arteries are systematically assessed following the protocol established by Logallo et al. [31]. A 5–1 MHz sector array probe (iU22 Philips Medical Systems, Bothell, WA, USA) is used for bilateral insonation of the sphenoidal segment (M1) and the insular segments (M2) of the middle cerebral arteries (MCA) in the axial planes. Peak systolic velocity (PSV) is measured from M1 origin to distal M2 segments with a 2 mm sample volume, by stepwise depth decrement, and stepwise optimal angle correction of Doppler sampling.

b) *Carotid artery ultrasonography*. The carotid arteries are examined by use of a 9–3 MHz linear array transducer (iU22 Philips Medical Systems, Bothell, WA, USA).

b1) Overview, stenoses, hemodynamics and velocities: For an orientating overview, initial B-mode scans in transversal and longitudinal plane are performed to visualize the common carotid artery (CCA), carotid bifurcation (BIF), and internal carotid artery (ICA). Segments of interest are stored as frozen images, or as video loop. Observation of carotid atherosclerotic plaques, stenosis, occlusion, dissection or fibromuscular dysplasia are noted. In case of stenosis, according to the international consensus statement [32], geometric lumen reduction is assessed by calculation of area reduction in the cross-sectional plane, using the combination of B-mode and color flow. Measurement of area reduction is considered independent from morphological configurations of the stenosis. Further, color and power Doppler modes are used for evaluation of hemodynamic effects in longitudinal plane, such as orthograde or retrograde flow, color aliasing phenomenon or turbulence. Blood flow velocities are measured using Pulsed-Wave (PW) Doppler under optimal angle correction. The maximum peak systolic velocity (PSV) is noted for the distal CCA, for the carotid bifurcation under visualization of the proximal ICA, and for the proximal ICA, of which frozen pictures are stored.

**Figure 2:**
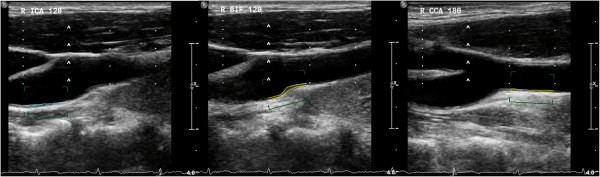
Ultrasonographic IMT measurement in the proximal ICA (left), the bifurcation (middle) and the distal CCA segment (right) by QLAB software.

**Figure 3:**
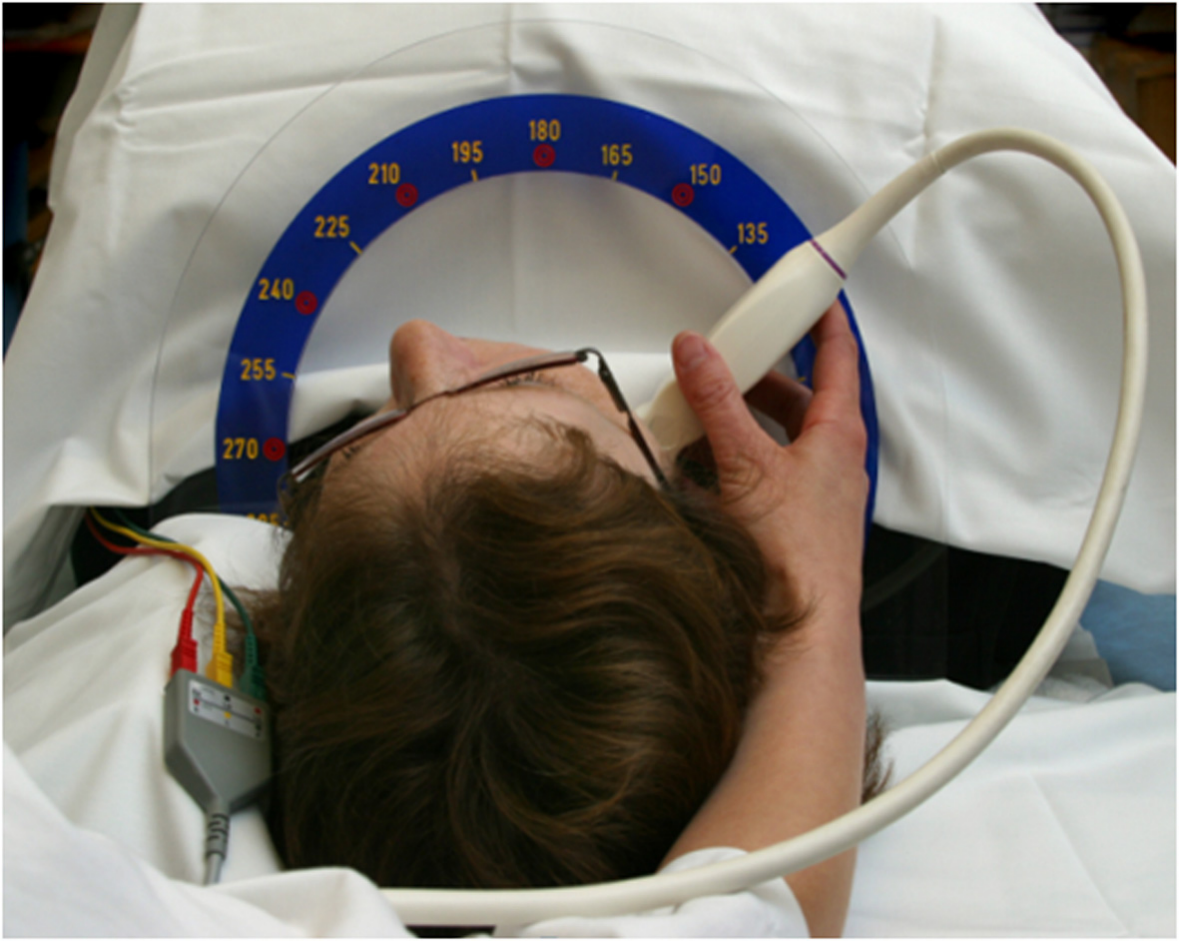
**Meijer’s Carotid Arc**® **(publication with written informed consent by the patient).**

b2) IMT-measurements: Vertical markers in a horizontal distance of 10 mm each are used to define the distal CCA, the bifurcation (BIF), and the proximal ICA segment in longitudinal view, using the tip of the flow divider (TFD) as internal landmark for placement of the second vertical marker (Figure 2). The CCA segment is defined 20–10 mm proximally to the TFD, the BIF segment is defined as 10–0 mm proximally to the TFD, and the ICA segment is defined as 0–10 mm distally to the TFD. Intima-media thickness (IMT) is visualized in longitudinal view on the far and, if possible, on the near wall of each segment, to ensure center position of the scan plane in the artery. Meijer’s Carotid Arc® (Figure 3) is used for standardization of the scan angles, performed at 180°, 150°, 120° and 90° in the right CCA segment, and at 180°, 210°, 240°, and 270° in the left CCA segment. IMT-measurements in BIF and ICA segments are bilaterally performed at the angle representing the most significant pathological finding, respectively. All measurements are performed in the enddiastolic phase of the cardiac cycle. Frozen pictures are stored for each measurement. IMT-analysis is performed by Philips QLAB-software after completed examination. IMT measurements are done over a distance of 10 mm for each far wall segment (Figure 2), and are stored as a mean value. In case of irregular IMT or presence of plaques, measurements of the maximum IMT or plaque thickness are additionally performed. Due to updated Mannheim Carotid Intima-Media Thickness Consensus criteria, plaques are defined as focal IMT measurements >1.5 mm [33]. Plaque surface is being evaluated as smooth, irregular or ulcerated.

**Figure 4:**
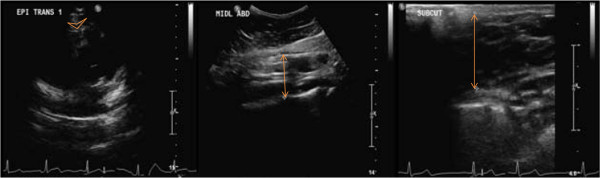
Ultrasonographic epicardial (left), intraabominal visceral (middle) and abdominal subcutaneous adipose tissue (right) in B-mode ultrasonography.

**Figure 5:**
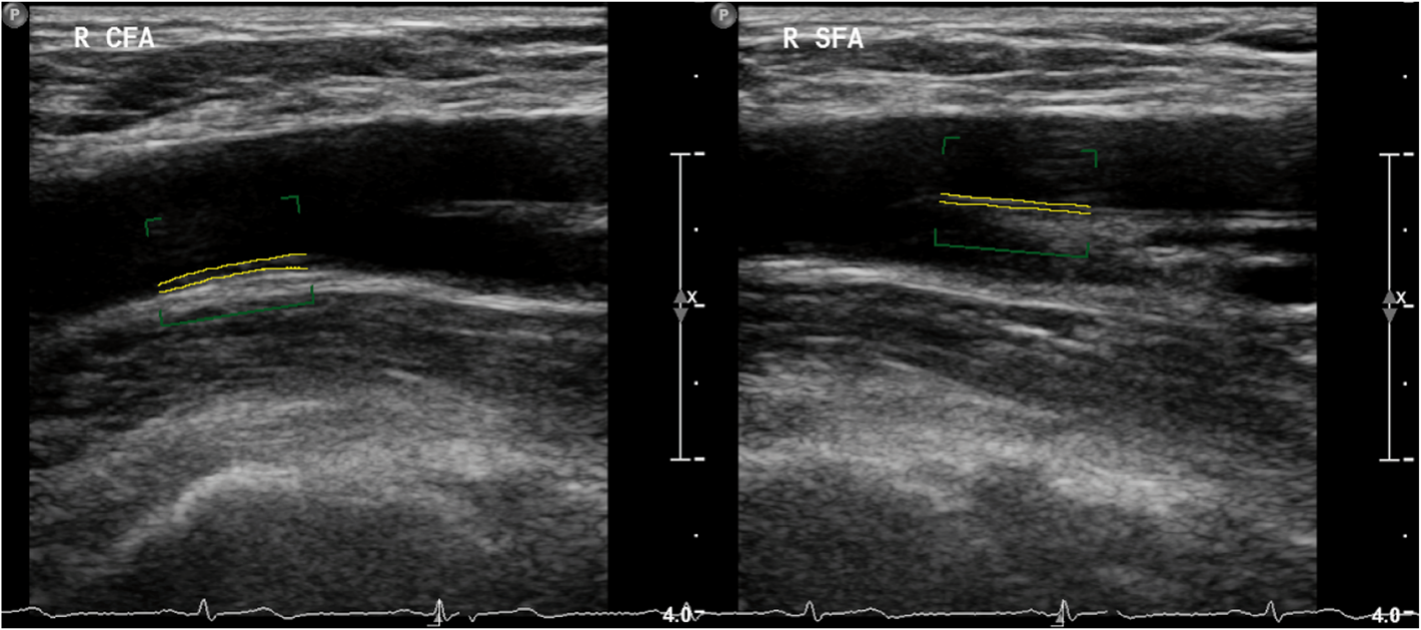
B-mode ultrasonography and IMT measurement in the distal CFA (left) and the proximal SFA segment (right) performed by QLAB software.

c) *Ultrasonographic epicardial adipose tissue (EAT)*. EAT measurements are performed by use of a 5–1 MHz sector array transducer (iU22 Philips Medical Systems, Bothell, WA, USA). The subject is placed in left lateral decubitus position. Epicardial fat thickness is measured on the free wall of the right ventricle from parasternal short-axis view during end-systole. EAT is defined as the echo-free space between the outer layer of the myocardial wall and the visceral layer of the pericardium [34,35] (Figure 4). The mean of three maximum value measurements is calculated.

d) *Ultrasonographic visceral abdominal adipose tissue (VAT)*. VAT measurements are performed by use of a 5–1 MHz curved array transducer (iU22 Philips Medical Systems, Bothell, WA, USA). All measurements are performed in longitudinal view on umbilicus level, and the distance between the external face of the rectus abdominis muscle/the peritoneum and the lumbar spine is used [36] (Figure 4). All measurements are performed at the end of expiration and without distortion of the abdominal cavity due to compression. The vertebral column is positioned horizontally. VAT is measured in frontal median position, 10 cm laterally to the left and 10 cm laterally to the right for the median position, and the mean value of these three measurements is calculated.

e) *Ultrasonographic subcutaneous abdominal adipose tissue (SAT)*. SAT measurement is performed by use of a 9–3 MHz linear array transducer (iU22 Philips Medical Systems, Bothell, WA, USA).The transducer is positioned transverse 1 cm above the umbilicus. SAT is defined as the distance between the cutis and the external face of the rectus abdominis muscle tendon plate (linea alba) (Figure 4), and is measured under maximum decompression of the skin.

f) *Abdominal aorta ultrasonography*.The abdominal aorta is examined by use of a 5–1 MHz curved array transducer (iU22 Philips Medical Systems, Bothell, WA, USA). Infrarenal vessel lumen is measured in longitudinal view, and external diameter measurement is repeated in transversal view. Infrarenal diameter >30 mm is suspect for aneurysm and considered for additional vascular surgical investigations [37]. Hemodynamically significant stenosis is assumed when PSV is ≥ 200 cm/s. Severity of atherosclerotic lesions is evaluated.

g) *Femoral artery ultrasonography*. The femoral arteries are examined by use of a 9–3 MHz linear array transducer (iU22 Philips Medical Systems, Bothell, WA, USA).Transversal view is used for identification of the common femoral artery (CFA) and localisation of the femoral artery bifurcation. Femoral IMT (fIMT) measurements are bilaterally performed in longitudinal view over a distance of 10 mm in the distal CFA (Figure 5) and in the proximal 10 mm of the superficial femoral artery (SFA), respectively (Figure 5). Frozen pictures are stored for each measurement. As for cIMT, fIMT is analyzed by Philips QLAB-software after completed examination, and performed over a distance of 10 mm for each far wall segment and stored as a mean value. In case of irregular IMT or presence of plaques, measurements of the maximum IMT and plaque thickness are additionally done.

h) *Ankle Brachial Index (ABI)*. ABI is measured after a resting period of at least 5 to 10 minutes by Ultrasonic Doppler Flow Detector, Model 811-BTS, Parks Medical Electronics, Inc., Aloha, OR, USA. Bloodpressure measurements are performed bilaterally in the radial, the dorsalis pedis, and the posterior tibial artery. ABI ≤ 0.9 at rest is defined as the cut-off point for peripheral artery disease (PAD). ABI 0.7-0.9 is considered as mild, 0.4-0.7 as moderate and <0.4 as severe arterial disease. ABI >1.4 may be explained by medial sclerosis or other conditions leading to arterial incompressibility [38]. Suspect subjects and participants with known diabetes mellitus are reported to the respective departments of vascular surgery for further investigation.

D Anthropometric variables, electrocardiography (ECG), and blood pressure measurements. Current height and weight are measured and Body mass index (BMI) is calculated as indicator for nutrition and body fat. Waist-hip ratio (WHR) is calculated from the respective circumference measurements. Systolic and diastolic blood pressures are measured bilaterally in the subject’s upper arm after a resting period. ECG is performed in all actively participating subjects.

E Neuroradiology (patients only). Routine initial neuroimaging includes a cerebral CT scan and CT-angiography (CTA), and is performed in all patients with acute stroke symptoms at hospital admission. Magnetic resonance imaging (MRI) may be the method of first choice in some patients. In absence of contraindications, MRI including axial FLAIR, MR-angiography (MRA), diffusion-weighted imaging (DWI) and susceptibility weighted imaging (SWI) is performed within 24 hours after admission. Intra- and extracranial artery stenoses are described as minor (≤50%), moderate (51-70%) and severe (71-99% maximum actual area reduction (AAR)) or occlusion.

F Arterial stiffness measured by aplanation tonometry (patients only). Carotid-femoral pulse wave velocity is measured using aplanation tonometry (SpygmoCor, AtCor medical, West Ryde, Australia) operated by a trained technician following a standardized program with inborn quality control assessment. Pulse waves from the carotid and femoral arteries are obtained with the tonometer and the pulse wave velocity is calculated taking the distance between the two measure points into account. From the carotid pulse wave, central (aortic) blood pressure is estimated.

G Ambulatory blood pressure monitoring. Twenty-four hour ambulatory blood pressure measurement is performed using a non-invasive ambulatory blood pressure monitor Diasys Integra II (Novacor, Cedex, France), set to auscultatory mode. The device is mounted on the non-dominant arm with an appropriately sized cuff, and the patients are instructed to relax their arm when readings are initiated. Blood pressure is pre-set to be measured every 20 minutes during daytime and every 30 minutes during night-time, giving an average of 78 measurements per 24 hours. Daytime is defined as the fixed period between 7 a.m. and 22 p.m.. The recording is accepted when > 70% of the measurements are technically valid, and otherwise will be repeated.

H CCTA including CT of the thoracic aorta (patients only). CCTA and CT of the thoracic aorta is performed in those patients found to have plaques in the femoral arteries and/or pathological ABI. For ECG-triggered CT-scanning, a Siemens dual FLASH scanner (Siemens Somatom Definition FLASH; Erlangen, Germany) is applied. Due to administration of I-contrast agents, patients with reduced glomerular filtration rate (GFR < 30 mL/min/1.73 m^2^) are excluded. Calcium scoring of coronary arteries is assessed before administration of intravenous contrast, whereas, the lumen and wall of the coronary arteries as well as the occurrence of aorta pathology is evaluated after intravenous contrast administration.

I Study of biomarkers and genetic analyses. Samples of serum and EDTA-plasma are collected, processed, coded and stored at −80°C until analyzation for each participating subject. Analyses are scheduled after completion of the 5-years inclusion period. Biomarkers to be investigated will be determined at the time of analyses according to the most relevant biomarkers known at that time point. GWAS, exone sequencing or any newer technology, relevant and feasible at the time of genetic analyses, will be applied.

### Primary and secondary prevention strategies

Additionally to stroke treatment and secondary prevention in the patient population, all subjects are being evaluated concerning the presence and severity of established clinical and sub-clinical cardiovascular disease and modifiable vascular risk factors. An evaluation sheet is given to all patients at discharge, issues where improvement is recommended are pointed out and intervention is initiated as soon as possible during hospital stay. For family members, a short report is being sent to their respective general practitioner, in which clinical and anamnestic results are discussed and recommendations for intervention or further investigation are given. A modified Essen Stroke Risk Scale is applied for all participants [39].

### Prospective follow-up

During a standardized telephone interview one week after discharge performed by a study nurse, patients are asked to evaluate the information they received concerning their stroke, investigation results, and individual vascular risk factors, as well as their hospital stay in general. Three months follow-up is performed at the out-patient clinic and includes standardized questionnaires concerning recurrent ischemic events, seizures, pain, cognitive function, psychological disorders, tolerability of medication, quality of life, employment/education after the stroke, sick leave, as well as changes with respect to life styles and modifiable risk factors after discharge. Clinical and functional scoring by NIHSS, mRS, and Barthel index are performed, and weight and blood pressure measurements are repeated. One-year follow-up is performed by telephone interview as short standardized questionnaire update on changes concerning modifiable risk factors.

For long-term follow-up, examinations C. a-h and D. will be repeated after 5, 10 and 15 years or after 10 and 20 years from inclusion regarding patients and partners or their offspring, respectively.

### Study endpoints

Primary endpoints are death and documented cerebral, coronary and/or peripheral arterial events. Secondary endpoints are the long-term development or progression of atherosclerosis and the failure of therapeutic goal achievement (tobacco cessation, well-regulated bloodpressure, dyslipidemia and diabetes mellitus, and normal weight or slight overweight). Data validation will be done by medical record information.

### Statistics

All obtained data are registered in the NOR-SYS Research Registry. Statistical analyses are performed by’ STATA/SE for Windows’and ‘R’ in cooperation with a biostatistician.

## Discussion

Long-term follow-up studies of young stroke patients have shown high mortality and vascular morbidity compared to healthy controls [6-10,40]. Hence, a prospective cohort follow-up based on thorough investigation of clinical and sub-clinical vascular disease and risk factors is necessary in order to achieve a better long-term outcome.

Ultrasound imaging has been proved to be a sensitive and cost-effective method for the detection of arterial vessel disease in major arteries [16], as well as for the evaluation of adipose tissue [35,41]. For this reason, ultrasound was chosen as the predominating tool for the investigations in the NOR-SYS protocol. IMT increases are dependent on age, sex and cardiovascular risk [42]. However, the increase and prevalence of atherosclerotic lesions vary among different anatomical segments. Moreover, increased IMT has repeatedly been associated with cardiovascular risk factors and the incidence of cardiovascular events [19,43], and has been validated as a surrogate marker of atherosclerosis [44,45]. Atherosclerotic lesions are not distributed circumferentially, but develop asymmetrically [46], and their prevalence varies in the different artery segments [47]. In our study, Meijer’s Carotid Arc® is used for standardized imaging at defined angles [47,48], and cIMT and plaque measurements are aquired bilaterally in three carotid segments: the distal CCA, the bifurcation and the proximal ICA [47]. We suppose that this approach will improve the individual risk classification, as recently suggested [49]. It has also been suggested that the presence of carotid artery plaques may be even more representative for CVD prediction than increased cIMT itself [50]. Hence, plaque measurements are performed in addition to the standardized IMT measurements at all three carotid sites, if present.

Atherosclerosis is a systemic disease, and lesions are often to be found in several locations of the vasculature, such as in the peripheral arteries. Intermittent claudication is a frequent condition in western European populations [51,52] and associated with symptomatic CAD and cerebrovascular events [53,54]. Acute death due to PAD has been shown in 9% [40], compared to 45% and 42% due to cerebrovascular and coronary death, respectively [55]. The CFA has been reported as the segment most prone to IMT increase and plaque formation [42] compared to the SFA and the carotids. CFA IMT has beyond that been related to coronary angiographic [56] and echocardiographic parameters [57]. It is considered suitable for long-term observations concerning the natural development of atherosclerosis in healthy participants, and for the observation of treatment effects in a participant group requiring intervention [42]. For these reasons, IMT measurements are additionally performed bilaterally in the distal CFA and the proximal SFA segment, and included in study analyses. Atherosclerosis in the abdominal aorta is leading to aortic stenoses and PAD. Abdominal aortic aneurysms are also considered to be a manifestation of advanced atherosclerosis [58], and are frequently observed in patients with carotid stenoses, cardiovascular events and PAD [59]. Therefore, in NOR-SYS the abdominal aorta is evaluated with respect to atherosclerotic lesions, stenoses, and aneurysms. The ABI is performed in all participants as it is a well-established tool in investigation for peripheral artery disease and adds valuable information to vascular risk prediction [60,61].

Standard screening for a cardiac embolic source, including 24 hour heart rhythm registration and echocardiography is carried out in order to diagnose left ventricular hypertrophy, abnormal left ventricular geometry, and dilated left atrium as they are well-known predictors of stroke, both in the general as well as in the hypertensive population [62]. Blood pressure is measured after hospital discharge as an ambulatory 24-hour measurement as it has been proven to be closer associated with cardiovascular target organ damage and incident cardiovascular events than clinic pressure [63]. Ambulatory blood pressure measurements identify hypertension more accurately than clinic blood pressure measured during an acute stroke.

Measurement of arterial stiffness by carotid-femoral pulse wave velocity by aplanation tonometry may be useful in identifying arterial disease which is not captured by routine carotid ultrasound visualization [64].

NOR-SYS includes CCTA and CT of the thoracic aorta because of the well-known association between peripheral and coronary disease [65]. In addition, aortic arch atheroma or other wall disease of the ascending aorta or the aortic arch might cause the index-stroke or recurrent stroke [66].

Obesity is an increasingly prevalent disorder [67] which is associated with atherosclerosis and cardiovascular disease. Particularily abdominal obesity has been associated with metabolic syndrome [68], pre-clinical atherosclerosis [69], cardiovascular events [70] and mortality [70].

Epicardial adipose tissue (EAT) has its embryologic origin in common with mesenteric and omental fat, and all these are accordingly classified as visceral adipose tissue (VAT) [71,72]. Associations between VAT and cIMT [73], metabolic syndrome [74,75] and cardiovascular disease [76,77] have been reported in several studies. Release of free fatty acids due to the proximity to the portal circulation leading to direct lipotoxicity [78,79], and release of pro-inflammatory and pro-atherogenic cytokines and hormones with impact on endothelial function [80,81] are related issues. The accumulation of VAT has therefore been found to be an independent vascular risk factor, even within the normal range of BMI [82]. Accordingly, the anatomical relationship of EAT to the heart is providing local interaction with modulation of the coronary arteries and the myocardium, which may subsequently affect cardiac function and morphology [83-85]. On the other hand, subcutaneous adipose tissue, which is a non-portal fat type with less metabolic activity [86], has previously shown only a weak relationship to increased cIMT [73]. Its evaluation related to the amount of VAT and anthropometric parameters is assumed to be relevant for risk prediction and for that reason included in NOR-SYS. Anthropometric parameters such as BMI and WHR are simply applicable clinical tools and widely used in obesity evaluation. They are as well associated with ultrasonographic visceral adipose tissue measurements [41,87], and applied in NOR-SYS.

In conclusion, the major objective of NOR-SYS is the standardized gathering of anamnestic, clinical, and biological data concerning life styles, medical history, and clinical and subclinical vascular disease at several sites of the vasculature including body fat composition and anthropometric measurements in young ischemic stroke patients and their families. Standardized questionnaires and standardized ultrasound examinations combined with detailed clinical data are assumed to increase the precision in diagnostics and risk estimation, and generate a solid basis of decision-making concerning secondary prophylaxis after acute ischemic stroke.

Further investigation and evaluation of vascular risk factors and sub-clinical artery wall disease in young ischemic stroke patients’ family members provide a platform for primary prophylaxis and early intervention.

NOR-SYS aims to reduce co-morbidity, disability, recurrent stroke, cognitive impairment and mortality in young patients with acute ischemic stroke. We expect that a comprehensive work-up and long-term observation, combined with biological, genetical and clinical information gathered from three family generations, will give the opportunity to improve our basic knowledge concerning preclinical atherosclerosis in families with a vascular disease burden.

NOR-SYS is reviewed and approved by the Regional Committee for Medical and Health Research Ethics, Western-Norway (REK-Vest 2010/74), and registered in ClinicalTrials.gov: NCT01597453.

## Competing interests

The authors declare that they have no competing interests.

## Authors’ contributions

All authors contributed to the study design in their respective professional field, helped to draft the manuscript, and read and approved the final manuscript.

## Pre-publication history

The pre-publication history for this paper can be accessed here:

http://www.biomedcentral.com/1471-2377/13/89/prepub
